# Facile, Efficient Copolymerization of Ethylene with Norbornene-Containing Dienes Promoted by Single Site Non-Metallocene Oxovanadium(V) Catalytic System

**DOI:** 10.3390/polym9080353

**Published:** 2017-08-11

**Authors:** Kaiti Wang, Jiabao Wang, Yanguo Li, Li Pan, Yuesheng Li

**Affiliations:** 1 Tianjin Key Lab of Composite & Functional Materials, School of Materials Science and Engineering, Tianjin University, Tianjin 300072, China; tkwang@ciac.ac.cn (K.W.); ysli@tju.edu.cn (Y.L.); 2 Changchun Institute of Applied Chemistry, Chinese Academy of Sciences, Changchun 130022, China; liyg@ciac.ac.cn; 3 National Center for Nanoscience and Technology, Beijing 100190, China; wangjb@nanoctr.cn

**Keywords:** cyclic olefin, copolymerization, diene, chain transfer, vinyl addition

## Abstract

Non-metallocene oxovanadium(V) complexes bearing either [ONNO]-type amine pyridine bis(phenolate) ligands or [ONN]-type amine pyridine phenolate ligands were employed as efficient catalysts to copolymerize ethylene with several unsymmetrical norbornene-containing dienes, such as 5-vinyl-2-norbornene (VNB), 5-ethylidene-2-norbornene (ENB) or dicyclopentadiene (DCPD), producing copolymers with high comonomer incorporations (VNB: 33.0 mol %; ENB: 30.4 mol %; DCPD: 31.6 mol %, respectively) and high molecular weight (VNB: 86.4 kDa; ENB: 256 kDa; DCPD: 86.4 kDa, respectively). The enchainment of the dienes was proven to be exclusive of vinyl-addition via the C=C double bond of the norbornene ring while the other double bond was retained near the backbone without crosslinking. During the copolymerization of ethylene with ENB, a positive ‘comonomer effect’ was observed. The catalytic activities of the catalysts as well as the molecular weights and comonomer incorporations of the resultant copolymers could be tuned within a wide range by varying the structures of the catalysts and copolymerization conditions. The [ONN]-type oxovanadium(V) complexes showed higher catalytic activities than those of [ONNO]-type oxovanadium(V) complexes, irrespective of the structure of the dienes. In addition, the dominant chain transfer pathway of the non-metallocene oxovanadium(V) catalytic system promoted copolymerization was proven to be transfer to aluminum compounds.

## 1. Introduction

The synthesis of well-defined polyolefin with reactive pendent vinyl group, which can be easily converted into functional groups by further chemical transformations, has drawn much attention over recent decades [1,2,3,4,5,6,7,8,9,10]. Among the various dienes successfully incorporated into the linear polyethylene (PE) or polypropylene (PP) main chain, the unsymmetrical dienes, such as *p*-(3-butenyl) styrene and dicyclopentadiene (DCPD), are very promising because of their two double bonds usually showing different selectivity toward coordinative incorporation and subsequent chemical transformation, respectively. The undesired crosslinking thus could be avoided and the microstructure of the resulting copolymers therefore might be well-controlled [11,12]. For instance, the cyclic olefin copolymers (COCs), which are usually synthesized from copolymerization of ethylene with cyclic olefin, are kinds of materials have higher modulus than high density polyethylene (HDPE) and PP, and exceptional moisture barrier for a clear polymer along with low moisture absorption rate. The COCs containing the reactive olefin groups are expected to provide functional COCs with substantially improved properties, and thus have attracted increasing attentions [13,14,15,16,17,18,19,20,21,22]. Several catalytic systems, including Ziegler–Natta catalysts [21], metallocenes [15] and non-metallocenes [13,14,16], have demonstrated good performance in the copolymerization of ethylene with various cyclic dienes.

**Scheme 1:**
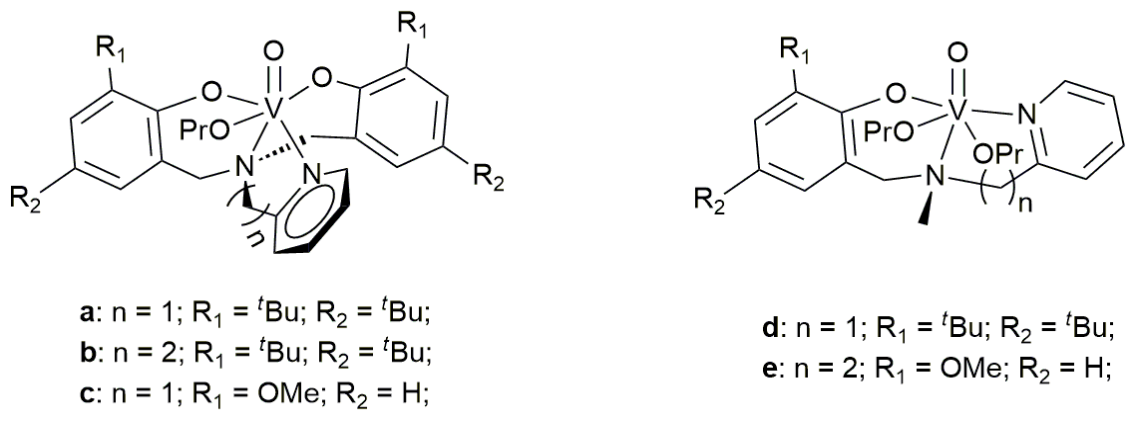
Structures of [ONNO]-type (**a**–**c**) and [ONN]-type (**d**,**e**) oxovanadium(V) complexes.

Among the classical Ziegler–Natta type catalysts, vanadium catalysts show outstanding catalytic properties for the manufacture of synthetic rubber and elastomers [23,24,25,26,27]. For example, Christman D.L. et al. reported that either VOCl_3_/Et_3_Al_2_Cl_3_ or VO(OtBu)_3_/Et_3_Al_2_Cl_3_ showed high cyclic dienes incorporation ability in the terpolymerization with ethylene and propylene [23]. Since the vanadium catalysts might be deactivated during the polymerization because of the reduction to low-valent, inactive species [27], introduction of bulky and polydentate ancillary ligands to stabilize active vanadium(V) species has been proven to be an effective approach to keep vanadium at high-oxidation and subsequently prolong the catalytic lifetime [28,29,30,31,32,33,34,35,36]. In our previous work, the synthesized [ONNO]-type and [ONN]-type oxovanadium(V) complexes also showed high catalytic activities and promising stabilities in copolymerization of ethylene with norbornene (NBE), and produced E/NBE copolymers with high NBE contents [37,38]. Their good performance prompts us to further investigate their capability of copolymerizing ethylene and norbornene derivatives with an additional C=C double bond, including 5-vinyl-2-norbornene (VNB), 5-ethylidene-2-norbornene (ENB), and DCPD. In this context, we presented the result of copolymerization of ethylene and above mentioned norbornene-containing comonomers by using several selected vanadium complexes with different ligand, including both [ONNO]-type (**a**–**c**) and [ONN]-type (**d**,**e**) oxovanadium(V) complexes (Scheme 1).

## 2. Materials and Methods

### 2.1. General Procedures and Materials

All air- and moisture-sensitive compounds were manipulated using standard Schlenk techniques or in an MBraun glovebox (Shanghai, China) under a dry nitrogen atmosphere. All solvents used were purified from an MBraun SPS system (Shanghai, China). Ethyl trichloroacetate (ETA) were purchased from Aldrich (Shanghai, China), dried over calcium hydride at room temperature and then distilled. VO(OnPr)_3_, phenol compounds, and amine compounds were purchased from Aldrich (Shanghai, China). Diethylaluminium chloride was obtained from Albemarle Corp (Charlotte, NC, USA). Commercial ethylene was directly used for polymerization without further purification. VNB, ENB, and DCPD were purchased from J&K Scientific Ltd. (Beijing, China), dried over calcium hydride at room temperature and then distilled under reduced pressure. The oxovanadium(V) complexes **a**–**e** were synthesized according to our previously reported procedures [37,38].

### 2.2. Characterization

The NMR results of polymers were obtained through a Bruker 400 MHz spectrometer (Beijing, China) at 120 °C with *o*-C_6_D_4_Cl_2_ as the solvent. The molecular weights and the polydispersities of the polymer samples were determined at 150 °C by use of a PL-GPC 220 type high-temperature chromatograph (Beijing, China) equipped with three PL gel 10 μm Mixed-B LS type columns. 1,2,4-Trichlorobenzene (TCB) was employed as an elute solvent at a flow rate of 1.0 mL/min. The calibration was made by the polystyrene standard Easi Cal PS-1 (PL Ltd., Beijing, China).

### 2.3. General Procedure for Copolymerization

The copolymerizations were carried out in a mechanically stirred 200 mL stainless steel autoclave, equipped with an electric heating mantle controlled by a thermocouple dipping into the reaction mixture. The autoclave was baked under vacuum for 4 h at 150 °C and then thermostatted to the desired temperature. The prescribed amounts of toluene, comonomer (ENB, VNB, or DCPD), Et_2_AlCl, ETA, and a toluene solution containing vanadium complex were added. Additional toluene was injected into this system via a syringe to keep a total volume of 50 mL. Subsequently, the reaction mixture was pressurized to the prescribed ethylene pressure and maintained throughout the polymerization. After a desired time, the stirring motor was stopped, the reactor was vented, and the resultant mixture was poured into EtOH/HCl (95/5). The resultant polymer as white precipitate was collected by filtration, adequately washed with EtOH and dried under vacuum at 60 °C until a constant weight was achieved.

## 3. Results and Discussion

### 3.1. Copolymerization of Ethylene with VNB

The copolymerization of ethylene with VNB was carried out at 50 °C under 4 atm of ethylene pressure. The typical copolymerization results were summarized in Table 1. Although the catalytic activities of the copolymerization are lower than those of ethylene homopolymerization under similar reaction conditions, both [ONNO]-type oxovanadium(V) complexes and [ONN]-type oxovanadium(V) complexes showed pretty good performance in the copolymerization of ethylene with VNB in toluene under mild conditions. Moreover, the [ONN]-type oxovanadium(V) catalysts **d** and **e** showed much higher catalytic activities than those of the [ONNO]-type analogues (complexes **a**–**c**), owing to their less steric hindrance around the metal center (entries 2, 4, and 6 vs. 16 and 18, Table 1). However, the copolymers produced by the latter possessed higher molecular weights (MWs). All the resultant copolymers exhibited unimodal molecular weight distribution, suggesting the single site catalytic behaviors of these catalysts. Among these three [ONNO]-type oxovanadium(V) complexes, complex **b** showed the highest catalytic activities in both homopolymerization of ethylene and copolymerization because of its less steric hindrance originated from the longer bridge between the pendant pyridine donor and the ligand skeleton, and produced copolymers with moderate MWs. Besides the reduction in catalytic activities, the MWs of the resultant polymers were decreased remarkably, and they were further decreased with the increase of VNB concentration in feed (entries 4, 9–14). This result indicated that, during the copolymerization, the continuous coordination-insertion process might be terminated by stable coordination of the pendant vinyl group with the active vanadium center and thus the chain growth was terminated. This was further proved by the ***N*_polym_*/N*_cat_** values showed in Table 1. The numbers of polymer chains produce by one catalytic molecule decreased as the VNB concentration in feed increased. A similar phenomenon has also been observed in the copolymerization of ethylene with VNB by using half-metallocene zirconium(IV) catalysts [39]. When the feedstock of the Et_2_AlCl was increased from 2000 to 6000, the MWs of the resulting copolymers obtained by complex **b** were also decreased (entries 4, 7, and 8, Table 1), and the number of polymer chains produce by one catalytic molecule increased, indicating that the chain transfer to aluminum compounds was the dominant chain transfer pathway during the copolymerization of ethylene with VNB.

**Figure 1:**
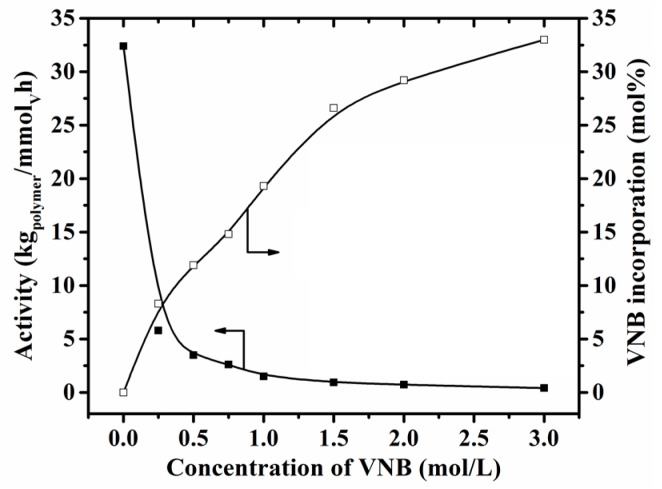
The influences of VNB concentration in feed on the catalytic activities of complex **b** and VNB incorporations in the copolymers.

With the same concentration of VNB in feed (0.5 mol/L), complexes **b** and **e** produced copolymers with higher VNB incorporations (11.9 and 10.4 mol %, respectively) than those of other catalysts. As shown in Figure 1, the VNB incorporation was increased steadily (from 8.3 to 33.0 mol %) with the increase of comonomer feedstock concentration (from 0.25 to 3.0 mol/L, entries 4, 9–14, Table 1), which means that the incorporation of VNB in the copolymers could be easily tuned in a wide range by adjusting the VNB feedstocks. Moreover, all the copolymers with high VNB contents were completely soluble in toluene at 50 °C with undetectable crosslinking.

**Figure 2:**
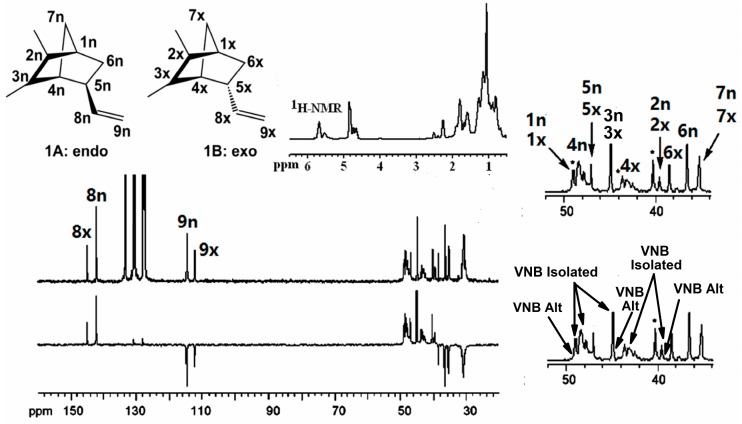
^13^C and ^1^H NMR spectra of E/VNB copolymer with VNB incorporation of 33.0 mol % produced by **b**/Et_2_AlCl/ETA catalytic system (Entry 14 in Table 1).

The incorporations and stereoselectivity of VNB were unambiguously revealed by ^1^H, ^13^C, and DEPT NMR spectra. All resonances were clearly identified and the data agreed well with the previously reported values [15,16]. No signals around 6.1 ppm relative to the C=C double bond of norbornene presented, while the resonances between 4.8 and 6.0 ppm corresponding to the pendant vinyl groups could be observed in the ^l^H NMR spectra of the copolymers. The enchainment of VNB was therefore proven to be exclusively via 2, 3 vinyl-addition of the norbornene ring. The insertion manner was also supported by the ^13^C NMR spectra of the copolymers, which presented the signals of pendant vinyl groups at 144.89 (=CH, *exo*), 142.18 (=CH, *endo*), 114.85 (=CH_2_, *endo*), and 112.09 ppm (=CH_2_, *exo*), respectively, with no detectable signals of the C=C double bond of norbornene around 136.9 ppm. According to the ^13^C NMR spectra, we could draw a conclusion that the incorporated VNB units showed two stereo-configurational structures in the E/VNB copolymer chains (1A endo and 1B *exo*, respectively, Figure 2). The chemical shifts of carbon 6 in ^13^C NMR spectra was significantly influenced by the *endo*/*exo*-configuration (6n: *endo*; 6x: *exo*, respectively), while the chemical shift of bridge carbon 7 was not affected. In addition, only the signals assigned to isolated VNB units and E/VNB alternating sequences were observed in the ^13^C NMR spectra. Even at high VNB concentration in feed, the signals of VNB continuous sequences were undetectable. This result is consistent with the fact that no homopolymer was obtained in homopolymerization of VNB by these catalytic systems.

### 3.2. Copolymerization of Ethylene with ENB

**Figure 3:**
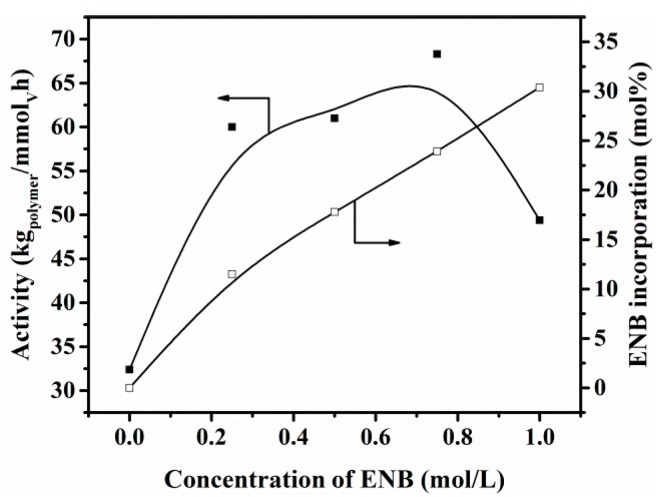
The influences of ENB concentration in feed on the catalytic activities of catalyst **b** and ENB incorporations in the E/ENB copolymers.

The copolymerizations of ethylene with ENB were also carried out under similar reaction conditions. Different with the phenomenon of copolymerization of ethylene and VNB, a “positive comonomer effect” was observed when the ENB feed concentration was enhanced [40,41]. As observed in Table 2, both [ONNO]-type oxovanadium(V) complexes and [ONN]-type oxovanadium(V) complexes showed much higher catalytic activities in the copolymerization than those of ethylene homopolymerizations (entries 3, 5, 15, and 17 in Table 1). The catalytic activities of catalyst **b** first enhanced with the increase of the ENB feedstock, achieved the maximum value of 68.3 kg/mmol_v_ h at 0.75 mol/L of ENB in-feed concentration (Figure 3). Copolymers with high MWs (101–256 kDa) and moderate to high ENB incorporations (11.5–30.4 mol % of ENB content, entries 1, 7–9, Table 2) were obtained. With the feedstock of Et_2_AlCl increasing, the *M*_W_s of the obtained copolymers decreased and the number of polymer chains produce by one catalytic molecule increased (entries 1, 3, and 4, Table 2), indicating that the dominant chain transfer pathway in E/ENB copolymerization was also transfer to aluminum compounds. Additionally, the resultant E/ENB copolymers also exhibited unimodal distribution originating from the single site catalytic behavior. Polymerization temperature and reaction time as significant reaction parameters were also explored. Both the catalytic activities and incorporation increased with reaction temperature, whereas the MWs of the copolymers decreased (entry 1 vs. 5, Table 2). This may be explained by the fact that higher reaction temperature accelerates both chain propagation and chain transfer. Neither the MW value nor ENB incorporation changed much with prolongation of reaction time from 5 min to 10 min (entry 1 vs. 6, Table 2). Similar to the copolymerization of ethylene with VNB, the [ONN]-type oxovanadium(V) complexes also showed higher catalytic activities than the [ONNO]-type oxovanadium(V) complexes in E/ENB copolymerizations, while the resultant copolymers produced by [ONNO]-type oxovanadium(V) complexes showed higher *M*_W_s as well (entries 1 and 2 vs. 10 and 11, Table 2).

As compared in Table 2, not only the catalytic activities of these oxovanadium complexes, but also the *M*_W_s of the obtained E/ENB copolymers were enhanced about one order of magnitude than those of the corresponding ethylene homopolymerizations. These results might benefit from the proper steric hindrance around the ethylidene group in ENB molecules. The coordination of the pendant ethylidene group to the central metal, which might result in deactivation of the catalysts, would be suppressed by its steric effect [39]. The prepared E/ENB copolymers by these oxovanadium(V) catalysts possessed much higher comonomer contents than those of the E/VNB copolymers. When the ENB concentration in feed was 0.5 mol/L, the comonomer incorporations were up to 16.9–18.8 mol %, irrespective of which oxovanadium(V) catalysts was employed. The ENB incorporation up to 30.4 mol % was achieved by increasing the in-feed concentration of ENB to 1.0 mol/L (Entry 9, Table 2). In addition, as observed, the increase of Al/V molar ratio led to an evident increase in both activity and comonomer incorporation (entries 1, 3, and 4, Table 2).

**Figure 4:**
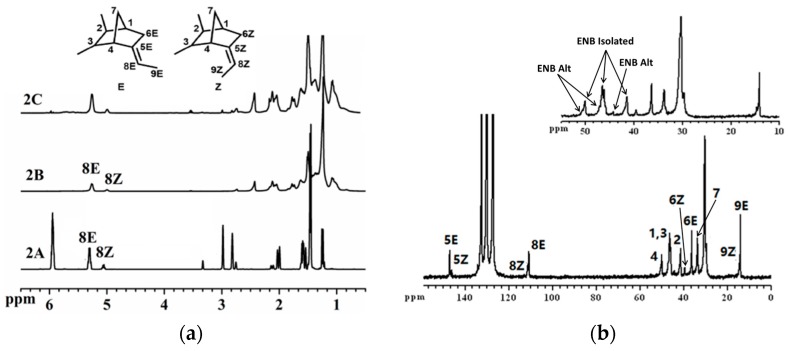
(**a**) ^1^H NMR spectra of ENB monomer (2A), E/ENB copolymer with 17.8 mol % of ENB content (2B, Entry 1 in Table 2) and E/ENB copolymer with 30.4 mol % of ENB content (2C, Entry 9 in Table 2), respectively; (**b**) ^13^C NMR spectrum of E/ENB copolymer prepared by **b**/Et_2_AlCl/ETA catalytic system (ENB incorporation: 30.4 mol %, Entry 9 in Table 2).

As shown in Figure 4a, the ^l^H NMR spectra of the E/ENB copolymers (2B and 2C) showed typical resonances at 5.1 and 5.3 ppm assigned to the ethylidene groups. The absence of resonances around 6.00 ppm implied that ENB inserted into the polymer chain exclusively via its intra cyclic C=C double bonds while the pendant C=C bonds remained. The E/Z configurational isomers mole ratio of 5:1 of the ENB monomer was also revealed and presented in Figure 4a [42]. This value is consistent with that of the raw ENB monomer (2A in Figure 4a), and indicates that both the two ENB isomers show the same reactivity toward these vanadium catalysts. The microstructure of copolymer sample 2C (entry 9, Table 2) was also investigated by ^13^C NMR spectrum (Figure 4b). Similar to the E/VNB copolymer, the microstructures of the copolymer produced by complex **b** only possessed isolated ENB inserted units and E/ENB alternating sequence. The resonances assigned to continuous ENB insertion were not observed.

### 3.3. Copolymerization of Ethylene with DCPD

**Figure 5:**
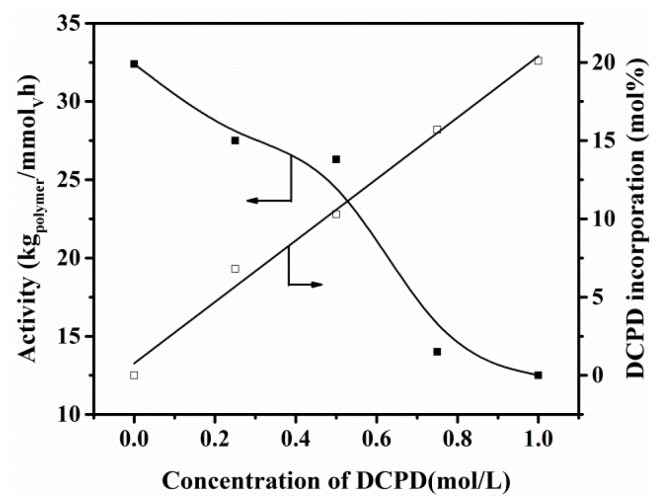
The influences of DCPD concentration on the catalytic activities of catalyst **b** and DCPD incorporations of the E/DCPD copolymers.

The copolymerizations of ethylene with DCPD catalyzed by these selected oxovanadium(V) complexes were also conducted in an efficient manner as well. Compared with the ethylene homopolymerization behavior, all of these oxovanadium catalysts showed slightly lower catalytic activities in E/DCPD copolymerizations, and the activities further decreased as the concentration of DCPD increased (Figure 5). It was revealed that the increase in comonomer concentration in feed is an effective approach to enhance DCPD incorporations in the resulting copolymers. When the concentration of DCPD was 1.00 mol/L, the DCPD content in the resultant copolymer was up to 31.6 mol %. In comparison with [ONNO]-type oxovanadium(V) complexes, the [ONN]-type analogues not only displayed much higher catalytic activities, but also yielded copolymers with higher DCPD contents (entries 1 and 8 vs. entries 11 and 12, Table 3). These phenomena should be ascribed to the less steric hindrance of the latter complexes which could allow the bulky DCPD comonomer to incorporate easily. The catalytic activities of these selected catalysts toward E/DCPD copolymerization and the *M*_W_s of resultant copolymers were lower than those of E/ENB copolymers, while higher than those of E/VNB copolymers. This result further demonstrated that the reactivity of the comonomer was influenced greatly by the steric environment around the unincorporated C=C bond. The Et_2_AlCl feedstock showed negligible influences on both the catalytic activities of oxovanadium(V) catalysts and the DCPD incorporations of the resultant copolymers, while the *M*_W_s of the copolymers decreased and the number of polymer chains produced by one catalytic molecule increased as the Al/V molar ratio raised from 2000 to 6000. These phenomena signified that the chain transfer to aluminum compounds was also the dominant chain transfer pathway in E/DCPD copolymerization.

**Figure 6:**
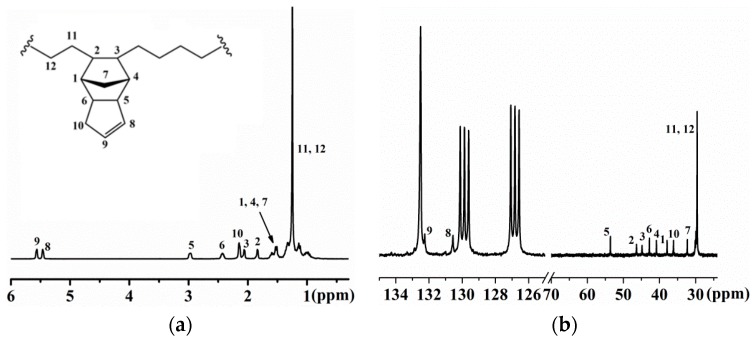
(**a**) ^1^H spectrum and (**b**) ^13^C spectrum of E/DCPD copolymer with DCPD of 15.7 mol % (entry 7 in Table 3) prepared by **e**/Et_2_AlCl/ETA catalytic system.

The microstructures of E/DCPD copolymers were also investigated by ^1^H and ^13^C NMR spectra (Figure 6). The signals at 5.53 and 5.65 ppm in ^1^H NMR spectrum as well as the peaks at 132.8 and 130.6 ppm in ^13^C NMR spectrum were assigned to the unreacted C=C double bond of the cyclopentene ring. It demonstrated that the copolymerization of ethylene with DCPD was achieved through a selective enchainment of norbornene ring because of its higher ring strain. Other signals of typical ethylene/DCPD copolymers in ^13^C NMR spectrum were assigned according to the literature report [14]. No evidence of crosslinking was detected for all ethylene/DCPD copolymers. Similar to the E/VNB and E/ENB copolymers, there were only isolated DCPD units and E/DCPD altering sequences existing in the copolymer chains, no continuous DCPD inserted sequences were detected, even for copolymers with high a DCPD incorporation of 31.6 mol %.

## 4. Conclusions

In the presence of Et_2_AlCl as a cocatalyst and ETA as a reactivating agent, a series of oxovanadium(V) complexes were proven to be efficient catalysts for copolymerizing ethylene with norbornene-containing dienes including VNB, ENB and DCPD, and copolymers possessing high comonomer content (above 30 mol %) and high molecular weight (up to 256 KDa) were obtained. The unimodal distribution (*M*_W_/*M*_n_ < 2.3) of the resultant copolymers indicated the single site catalytic behaviors of these catalysts. Benefiting from its proper steric environment around the metal center, complex **b** not only exhibited high catalytic activities toward the copolymerization, but also produced copolymers with promising *M*_W_s and comonomer incorporations. The selected dienes inserted into the polymer chain via the enchainment of the C=C double bond of the norbornene ring, and the other double bond was retained without crosslinking. ENB showed the highest reactivity among the three selected dienes, and the comonomer incorporations as well as the *M*_W_s of E/ENB copolymers were much higher than the other two types of copolymers because of the steric environment around the pendant ethylidene group. The dominant chain transfer pathway during the copolymerizations was proven to transfer to aluminum compounds, and the catalytic activities of the catalysts as well as the MWs and the comonomer incorporations of the copolymers could be tuned in a wide range by varying the reaction conditions.

## Figures and Tables

**Table 1:** Ethylene/VNB copolymerization by oxovanadium(V) complexes.

Entry	Cat. (μmol)	Al/V (Molar Ratio)	VNB (mol/L)	Activity (kg/mmol_V_ h)	VNB Incorp. ^a^	*M*_w_^b^ (kDa)	PDI ^b^	*T*_g_^c^ (°C)	*N*_polym_*/N*_cat_^d^
1	**a** (2.0)	4000	0	3.36	-	113	1.9	-	5.00
2	**a** (2.0)	4000	0.50	0.54	8.9	86.4	2.0	52	1.00
3	**b** (2.0)	4000	0	32.4	-	193	2.1	-	28.0
4	**b** (2.0)	4000	0.50	3.48	11.9	59.1	2.2	54	9.80
5	**c** (2.0)	4000	0	6.00	-	135	1.8	-	7.40
6	**c** (2.0)	4000	0.50	1.86	9.1	33.6	2.0	52	9.20
7	**b** (2.0)	2000	0.50	2.82	12.5	62.1	2.1	55	7.60
8	**b** (2.0)	6000	0.50	3.81	11.6	42.8	2.0	53	14.0
9	**b** (2.0)	4000	0.25	5.79	8.3	67.6	2.0	51	14.0
10	**b** (2.0)	4000	0.75	2.61	14.8	35.9	1.9	56	12.0
11	**b** (2.0)	4000	1.00	1.50	19.3	25.7	1.9	74	9.70
12	**b** (2.0)	4000	1.50	0.93	26.6	10.9	1.8	82	14.0
13	**b** (2.0)	4000	2.00	0.72	29.2	6.42	1.8	88	1.90
14	**b** (2.0)	4000	3.00	0.41	33.0	5.12	1.9	91	1.40
15	**d** (1.0)	4000	0	17.8	-	220	3.3	-	13.0
16	**d** (1.0)	4000	0.50	8.22	8.7	30.4	2.1	51	45.0
17	**e** (1.0)	4000	0	58.3	-	62.4	2.3	-	156
18	**e** (1.0)	4000	0.50	9.24	10.4	34.6	2.2	53	45.0
19	**e** (1.0)	4000	1.00	5.16	17.9	31.0	1.9	73	28.0

Polymerization conditions: the copolymerizations were carried out in 50 mL of toluene at 50 °C under 4 atm of ethylene pressure, the polymerization time was 10 min, ETA (300 equiv. to vanadium complexes) was employed as reactivating agent; ^a^ VNB incorporations were (mol %) calculated from ^1^H NMR spectra; ^b^ Weight-average molecular weight (*M*_w_) and polydispersity indexes (PDI) of the copolymers were determined by high temperature GPC at 150 °C in 1,2,4-trichlorobenzene; ^c^ The glass transition temperatures of the copolymers were determined by DSC; ^d^ The number of polymer chains produced by one catalytic molecule, which was calculated by the follow formula: (Mass of polymer/MW of polymer)/Molar of catalyst.

**Table 2:** Ethylene/ENB copolymerization by oxovanadium(V) complexes.

Entry	Cat. (μmol)	Al/V (Molar Ratio)	VNB (mol/L)	Activity (kg/mmol_V_ h)	ENB Incorp. ^a^	*M*_w_^b^ (kDa)	PDI ^b^	*T*_g_^c^ (°C)	*n*_polym_*/n*_cat_^d^
1	**b** (0.25)	4000	0.50	61.0	17.8	168	2.0	73	3.00
2	**c** (0.25)	4000	0.50	8.16	18.1	239	2.1	73	0.30
3	**b** (0.25)	2000	0.50	25.9	17.2	221	2.1	70	1.00
4	**b** (0.25)	6000	0.50	64.3	18.5	149	1.9	75	3.60
5 ^e^	**b** (0.25)	4000	0.50	48.5	15.3	256	2.2	63	1.60
6 ^f^	**b** (0.25)	4000	0.50	49.9	16.7	195	2.1	69	4.30
7	**b** (0.25)	4000	0.25	60.0	11.5	177	2.0	54	2.80
8	**b** (0.25)	4000	0.75	68.3	23.9	169	2.1	80	3.40
9	**b** (0.25)	4000	1.00	49.4	30.4	153	2.3	87	2.70
10	**d** (0.10)	4000	0.50	92.4	16.9	138	2.0	71	5.60
11	**e** (0.10)	4000	0.50	122	18.8	101	2.0	77	10.0

Polymerization conditions: the copolymerizations were carried out in 50 mL of toluene at 50 °C under 4 atm of ethylene pressure, polymerization for 5 min, ETA (300 equiv. to the catalyst) was employed as reactivating agent; ^a^ ENB incorporations (mol %) were calculated from ^1^H NMR spectra; ^b^ *M*_w_ and PDI of the copolymers were determined by high temperature GPC at 150 °C; ^c^ The glass transition temperatures of the copolymers were determined by DSC; ^d^ The number of polymer chains produced by one catalytic molecule, which was calculated by the follow formula: (Mass of polymer/MW of polymer)/Molar of catalyst; ^e^ Copolymerization was carried out at 25 °C; ^f^ Polymerization for 10 min.

**Table 3:** Ethylene/DCPD copolymerization by oxovanadium(V) complexes.

Entry	Cat. (μmol)	Al/V (Molar Ratio)	DCPD (mol/L)	Activity (kg/mmol_V_ h)	DCPD Incorp. ^a^	*M*_w_^b^ (kDa)	PDI ^b^	*T*_g_^c^ (°C)	*N*_polym_*/N*_cat_^d^
1	**b** (0.50)	4000	0.50	23.3	10.6	103	2.1	55	38.0
2	**c** (0.50)	4000	0.50	1.32	8.5	54.2	1.7	52	4.00
3	**b** (0.50)	2000	0.50	29.8	11.1	134	2.3	56	37.0
4	**b** (0.50)	6000	0.50	26.3	10.3	87.4	2.1	55	50.0
5 ^e^	**b** (0.50)	4000	0.50	24.7	9.7	96.8	2.0	52	21.0
6	**b** (0.50)	4000	0.25	27.5	6.8	131	1.9	51	35.0
7	**b** (0.50)	4000	0.75	14.0	15.7	89.4	1.9	65	26.0
8	**b** (0.50)	4000	1.00	12.5	20.1	63.1	1.6	77	33.0
9	**d** (0.25)	4000	0.50	39.4	19.8	80.9	2.0	76	81.0
10	**e** (0.25)	4000	0.50	50.9	21.4	71.4	2.2	79	119
11	**e** (0.25)	4000	1.00	32.9	31.6	59.7	2.1	98	92.0

Polymerization conditions: the copolymerizations were carried out in 50 mL of toluene at 50 °C under 4 atm of ethylene pressure, polymerization for 10 min, ETA (300 equiv. to vanadium complexes) was employed as reactivating agent. ^a^ DCPD incorporation (mol %) were calculated by ^1^H NMR spectra; ^b^ Weight-average molecular weight and polydispersity index of the copolymers were determined by high temperature GPC at 150 °C; ^c^ The glass transition temperatures of the copolymers were determined by DSC; ^d^ The number of polymer chains produced by one catalytic molecule, which was calculated by the follow formula: (Mass of polymer/MW of polymer)/Molar of catalyst; ^e^ Copolymerization for 5 min.
